# Supplementary material for: Conceptualising the Integration of Strategies by Clinical Commissioning Groups in England Towards the Antibiotic Prescribing Targets for the Quality Premium Financial Incentive Scheme: A Short Report
Source: Antibiotics (Basel). 2020 Jan 23;9(2):44. doi: 10.3390/antibiotics9020044 (PMC7167865; doi:10.3390/antibiotics9020044)
Supplement: Supplementary file 1 [file antibiotics-09-00044-s001.pdf]

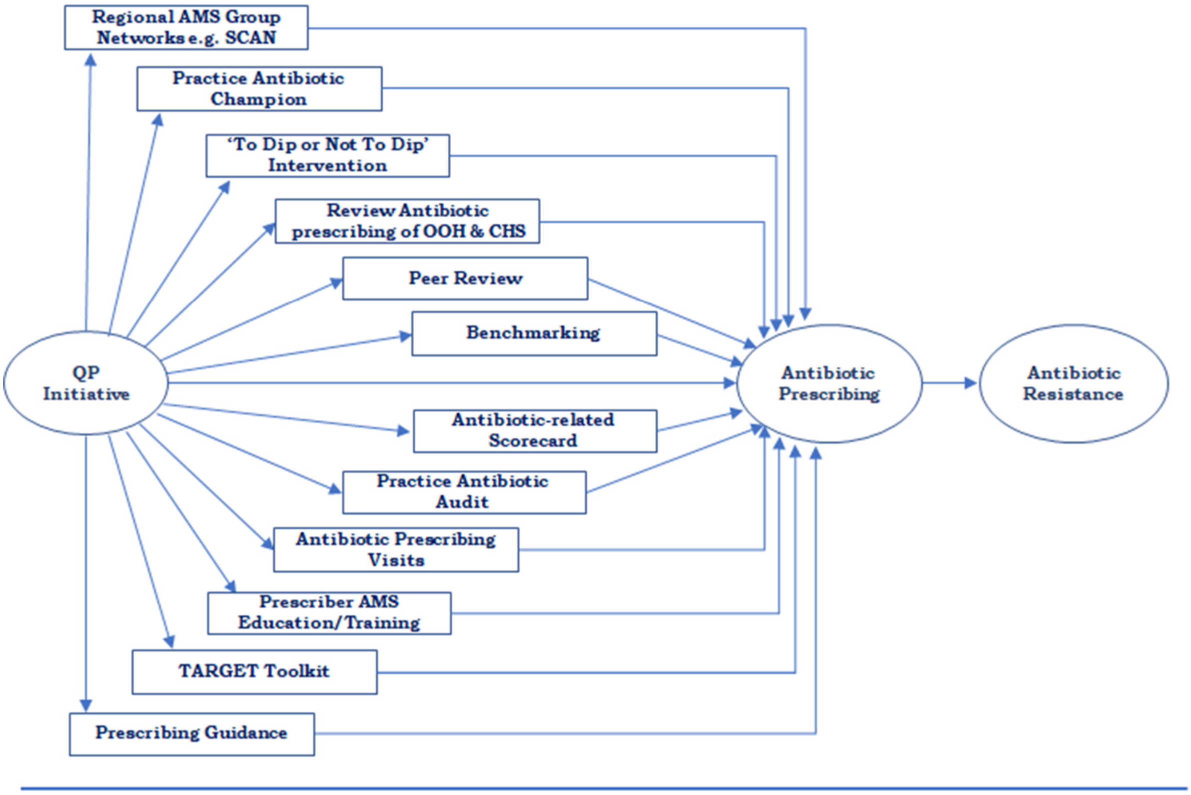

Supplementary Material 1:
Conceptual model developed by Group 1

Figure S1: Initial Conceptual Model Group 1

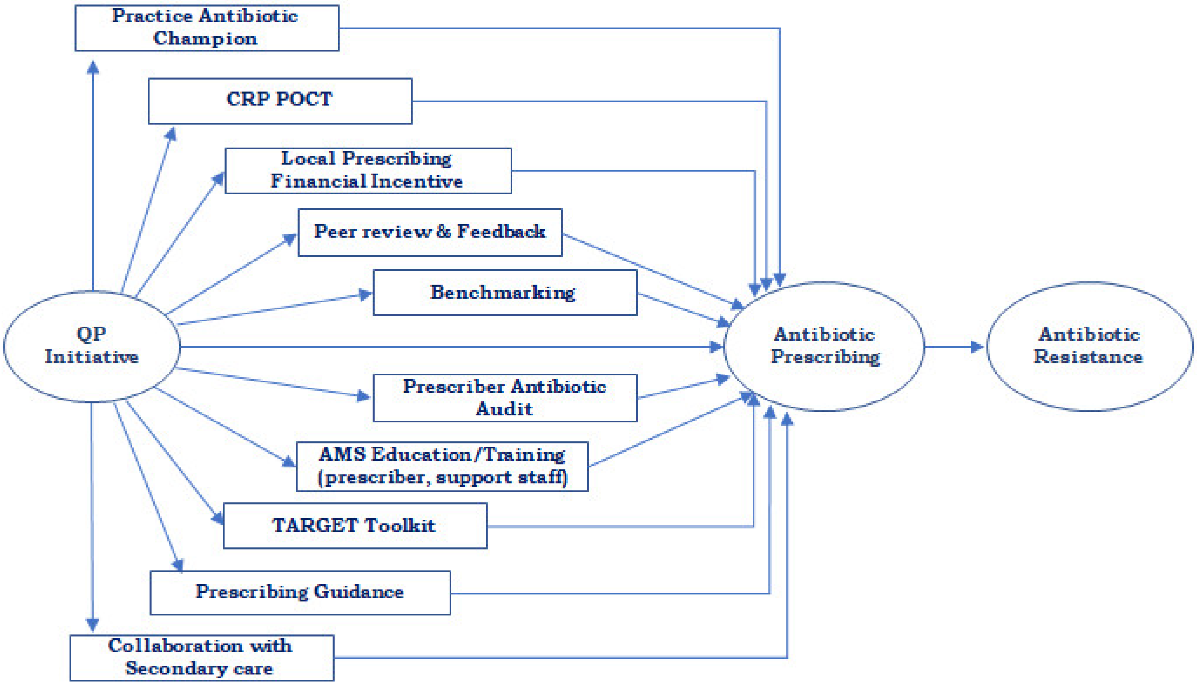

Supplementary Material 2:
Conceptual model developed by Group 2

, Figure S2: Initial Conceptual Model Group 2,

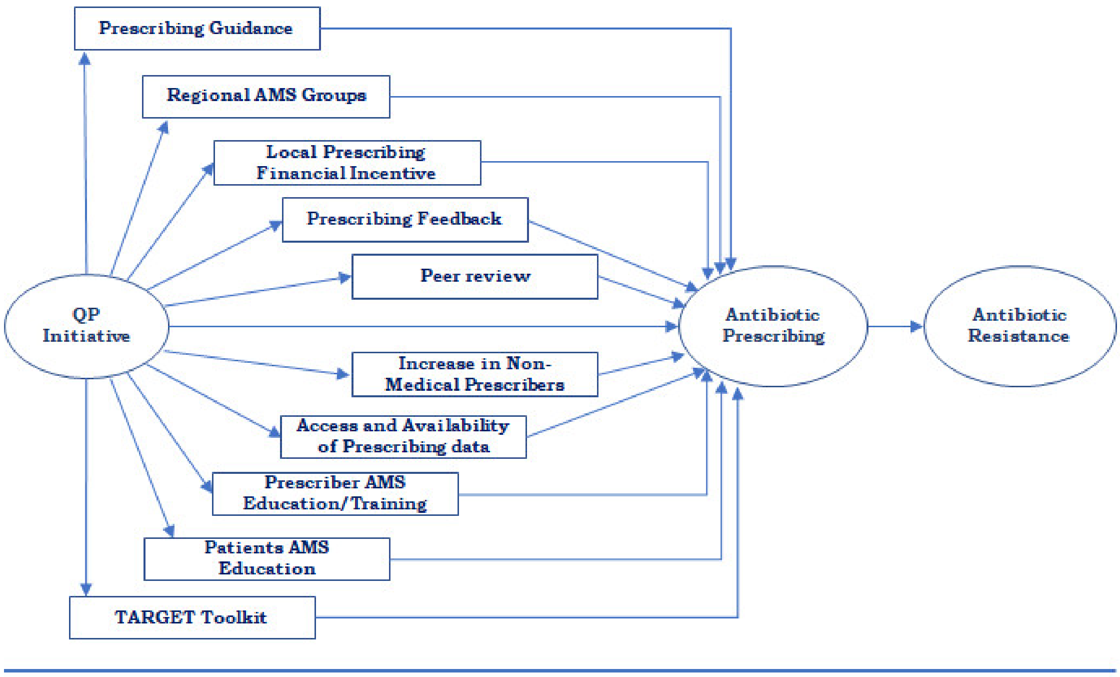

Supplementary Material 3:
Conceptual model developed by Group 3

Figure S3: Initial Conceptual Model Group
